# A Transition of Care Service to Reduce Hospital Readmissions for High-Risk Cardiology Patients (RECARD): Protocol for a Pre-Post Interventional Trial

**DOI:** 10.2196/94841

**Published:** 2026-09-25

**Authors:** Nazanin Falconer, Shelley Wilkinson, Centaine Snoswell, Chariclia Paradissis, Kelvin Robertson, Ian Coombes, William Y S Wang, Keshia De Guzman, Holly Foot, Liza-Jane Raggatt, Jared Miles, Vivian Bryce, Andrew Jones, Gloria Chui, Estelle Jensen, Sue Carson, John Atherton, Michael Barras

**Affiliations:** 1 School of Pharmacy and Pharmaceutical Sciences, The University of Queensland, 20 Cornwall St, Woolloongabba, Queensland, 4102, Australia, 61 061412342551; 2 Pharmacy Department, Princess Alexandra Hospital, Brisbane, Queensland, Australia; 3 Department of Obstetric Medicine, Mater Mothers' Hospital, South Brisbane, Queensland, Australia; 4 Townsville Hospital, Townsville, Queensland, Australia; 5 Royal Brisbane and Women's Hospital, Brisbane, Queensland, Australia; 6 Department of Cardiology, Princess Alexandra Hospital, Brisbane, Queensland, Australia; 7 Medical School, Faculty of Health, Medicine and Behavioural Sciences, The University of Queensland, Brisbane, Queensland, Australia; 8 Office of the Medical Director, Queensland Ambulance Service, Brisbane, Queensland, Australia; 9 Pharmacy Department, Royal Brisbane and Women's Hospital, Brisbane, Queensland, Australia; 10 Data Science Collaborative Research Platform, The University of Queensland, Brisbane, Queensland, Australia; 11 Queensland University of Technology, Brisbane, Queensland, Australia; 12 Department of Cardiology, Royal Brisbane and Women's Hospital, Brisbane, Queensland, Australia

**Keywords:** transition of care, medication safety, hospital readmission, pharmacy services, protocol

## Abstract

**Background:**

Transition of care (TOC) is a high-risk period for medication errors as patients move between multiple health care professionals, settings, and changing medication regimens. Ineffective communication systems further increase the likelihood of medication harm and hospital readmission. A TOC service with tailored, patient-specific support may help reduce these risks. Cardiovascular medicines significantly contribute to medication harm, and patients recovering from acute myocardial infarction (AMI) or cardiothoracic surgery (CS) are vulnerable due to complex regimens and frequent medication changes. Pharmacists, as medication experts embedded within cardiology teams, are well positioned to support these high-risk patients, facilitate safer transitions from hospital to home, and provide coordinated postdischarge follow-up.

**Objective:**

This study aims to evaluate the impact of a pharmacist-led interdisciplinary TOC service on reducing medication harm for cardiology patients post-AMI or CS.

**Methods:**

The study will be conducted in two phases: phase 1—intervention development will combine scoping review findings with stakeholder input to design an evidence-based, cocreated, culturally relevant intervention, underpinned by implementation science. A risk prediction model will be developed and validated to identify AMI/CS patients at high risk of medication-related unplanned readmission within 30 days. Phase 2: the RECARD (Reduce Hospital Readmissions for High-Risk Cardiology Patients) trial will use a pre-post design across 3 Australian tertiary hospitals (2 metropolitan and 1 regional) to compare a retrospective usual care patient cohort (referred to as “pre”) with a different prospective patient cohort (referred to as “post”) that includes both usual care and intervention AMI/CS patients receiving the TOC service. Only high-risk poststudy patients will receive the intervention TOC service, consisting of an individualized bundle of TOC activities to optimize medication management, while the remaining poststudy patients will receive usual care. A unique feature of the service is its capacity for hospital clinicians to conduct postdischarge home visits for patients needing additional support. The primary outcome for this study is medication-related 30-day hospital readmission. The analysis will consist of multivariable logistic regression. An economic analysis will be conducted to evaluate the costs associated with the intervention and medication-related readmissions. A comprehensive process evaluation will be conducted using the RE-AIM (Reach, Effectiveness, Adoption, Implementation, and Maintenance) framework to enable learnings from the trial to inform sustainable improvements in this field. A patient survey will be administered at the end of the trial to assess patient experience and elicit patient preferences using a discrete choice experiment.

**Results:**

The predata are currently being collected for patients discharged from the 3 hospital sites during the 12-month prestudy period. The postdata intervention began on December 10, 2025. As of May 27, 2026, a total of 343 patients have been recruited at the 3 study sites.

**Conclusions:**

The findings of this study will inform future implementation of a sustainable cardiac TOC model within the Australian health care setting.

## Introduction

### Background

The transition from hospital to primary care is a high-risk period for patients due to complex communication among multiple health care professionals, frequent medication changes, and complex care plans [1]. These factors increase the likelihood of medication harm and hospital readmission [2]. Implementing a structured transition of care (TOC) service with tailored support may help mitigate these risks [3,4].

Cardiovascular disease remains one of the leading causes of mortality in Australia and is managed with high-risk medications, such as anticoagulants and antiplatelets, that require careful titration [5,6]. These medications are a major contributor to medication harm, leading to medication-related hospital readmissions, morbidity, and mortality [5]. Associated complications include bleeding, acute kidney injury, hypotension-related falls, and electrolyte disturbances. Patients recovering from acute myocardial infarction (AMI) or cardiothoracic surgery (CS) are particularly vulnerable [6,7]. A previous study by the authors found that 6% (94/1564) of post–myocardial infarction patients were readmitted due to severe medication harm [5]. Another Australian study reported that 93% of cardiology unit discharges involved at least one medication-related problem, often linked to uncertainty about medications, drug interactions, and adverse effects [8].

Health inequities further exacerbate adverse cardiovascular outcomes. A dedicated TOC practitioner, trained in medication management, who can advocate for and provide timely navigation for patients as they return to the community remains a significant gap for some of these specialized high-risk patient groups [9]. For instance, in Queensland, hospital programs such as Better Cardiac Care for Aboriginal and Torres Strait Islander teams have improved aspects of medication management, such as ensuring medication supply and improving discharge communication [7,10-13]. Systemic inequities, treatment complexity, and limited access to culturally safe or patient-tailored services likely heighten risks, necessitating tailored, co-designed TOC interventions, as we propose.

Current health care systems are not adequately designed to support patients postdischarge, highlighting the need for a multifaceted approach to improve medication management at TOC [14]. Postdischarge care is often hindered by inadequate communication channels, a lack of early engagement with patients and carers, and difficulty scheduling follow-up with general practitioners (GPs) [15]. Up to 50% of patients lack a regular GP or community pharmacist, which further compromises continuity of care [15]. Given the medication burden and complex health care needs of the post-AMI and CS population, a formal TOC approach with a pharmacist leading an interdisciplinary team is vital to improving outcomes and reducing readmissions. Pharmacist-led TOC services have been shown to reduce readmissions and medication harm while maximizing quality of life for cardiovascular patients [2]. A hospital-initiated, pharmacist-led multidisciplinary approach facilitates improved medication management by encouraging patient and caregiver engagement, development of shared care plans, and collaboration with health care professionals [16]. While existing programs address heart failure and cardiac rehabilitation [17], no dedicated initiatives focus on postdischarge medication management for high-risk patients following an AMI or CS event.

Community-based medication review programs, such as Home Medication Reviews and Residential Medication Management Reviews, provide some support but are not tailored for patients recently discharged after an AMI or CS [18]. These programs are also underutilized by Aboriginal and Torres Strait Islander peoples and those in rural and remote areas and are not always available in a timely manner for immediate postdischarge care [9]. The current TOC model relies heavily on written communication to GPs, which may not be received in time for actions to be taken and is not consistently shared with community pharmacists for their active clinical involvement [19]. Geographic barriers and limited health care access further hinder timely follow-up, increasing the risk of medication harm. The members of a TOC team can act as patient advocates, ensuring effective communication and timely care coordination postdischarge. Pharmacists are uniquely positioned to perform this role for high-risk patients due to the medication misadventure risks they face during TOC.

The TOC service to RECARD (Reduce Hospital Readmissions for High-Risk Cardiology Patients) patients is a program that will codevelop, implement, and evaluate a patient-centered, pharmacist-led, interdisciplinary TOC service to reduce medication-related hospital readmissions in patients following AMI and CS. There is no existing evidence-based framework that systematically addresses medication harm at TOC for this cohort. There is an urgent need for a structured, patient- and team-centered TOC model that proactively identifies high-risk patients, engages them to co-design their care plans, ensures seamless communication among health care providers, and provides a home-based visit. This approach will integrate pharmacists into TOC teams that include nurses and Indigenous health workers to provide timely medication management support and improve patient outcomes.

### Trial Hypothesis

Compared with usual care, the implementation of the interdisciplinary pharmacist-led TOC intervention will result in a significant reduction in 30-day medication-related hospital readmissions, be positively received by patients, and be cost-effective.

### Aim

To evaluate the effectiveness of a pharmacist-led interdisciplinary TOC service in reducing medication-related hospital readmissions among cardiology patients following acute AMI or CS.

### Study Objectives

#### Primary Objective

The primary objective is to determine if an interdisciplinary pharmacist-led cardiology TOC service reduces the rate of 30-day medication-related hospital readmissions compared with usual care.

#### Secondary Objective

The secondary objective is to evaluate the impact of the TOC service based on key domains of the RE-AIM (Reach, Effectiveness, Adoption, Implementation, and Maintenance) framework [20], including the following:

- Reach: The target population by the absolute number, proportion, and representativeness of patients who are willing to participate in the RECARD TOC service.
  - Proportion of patients who engage with the TOC service: The proportion of patients who consent to and engage with the TOC service, compared with the broader population of patients with AMI/CS and the high-risk patients across the sites (Princess Alexandra Hospital [PAH], Royal Brisbane and Women's Hospital [RBWH], and Townsville University Hospital [TUH]).
- Effectiveness: The impact of the TOC service on secondary outcomes, including clinical effects, potential negative effects, and economic outcomes.
  - 30-day all-cause readmissions: All patients who are readmitted to the hospital for any presentation or reason within 30 days.
  - 60-day all-cause readmissions: All patients who are readmitted to the hospital for any presentation or reason within 60 days.
  - 90-day all-cause readmissions: All patients who are readmitted to the hospital for any presentation or reason within 90 days.
  - Subanalysis of the primary outcome by cohort: 30-day medication-related readmission subanalysis for post-AMI patients and CS patients as subgroups.
  - Subanalysis of the primary outcome by site: 30-day medication-related readmission for metropolitan versus regional sites.
  - Subanalysis of the primary outcome by intervention: 30-day medication-related readmission for high-risk patients who received the intervention (TOC service) and those who did not, as subgroups.
  - Preventable harm: Preventability of 30-day medication-related readmissions as assessed through the modified Schumock and Thornton criteria [21].
  - Severity of harm: Severity of 30-day medication-related readmissions as defined through severity definitions by Morimoto et al [22].
  - Patient experience: Patient experience and preferences assessed through a survey.
  - Cost-effectiveness: Economic evaluation to assess the cost per readmission avoided.
- Adoption: The target staff, settings, systems, and communities by the absolute number, proportion, and representativeness of settings and staff willing to initiate and deliver the TOC service.
  - TOC staff field notes during service delivery: TOC service staff (ie, TOC pharmacist, TOC nurse, or Indigenous health care worker) notes on their experiences with delivering the service to post-AMI/CS patients.
  - Postimplementation interviews with TOC staff: Interviews undertaken with TOC service staff to determine their adoption of the service and willingness to deliver the service to patients.
- Implementation: Consistency, costs, and adaptations made during the delivery of the TOC service, both at the setting and individual levels.
  - Communication outcome (discharge summary): Number of discharge summaries provided to primary care providers.
  - Communication outcome (home visits): Number of follow-up home visit consultations provided within the TOC service.
  - Process outcome (adaptations to the TOC service): Adaptations made to the TOC service by TOC staff during the study, as indicated through the delivery of activities in the TOC bundle and relevant notes during service delivery.
- Maintenance (and Sustainability): Long-term cost-effectiveness and local policy and procedure development.
  - Economic sustainability: Sustainability of the intervention in the long-term based on the cost-effectiveness data.
  - Local policy and procedures: Local policies and procedures have been developed during the pilot that will support long-term sustainable adoption of the TOC model.

## Methods

This protocol was developed in accordance with the SPIRIT (Standard Protocol Items: Recommendations for Interventional Trials) statement (Checklist 1) [23].

### Design and Setting

The study will be conducted in 2 phases: the development of the intervention using co-design principles (phase 1) and the pre-post interventional study (RECARD trial; phase 2).

#### Phase 1: Trial Development

Phase 1 will be conducted in 2 parts, A and B.

##### Phase 1 Part A: Evidence and Stakeholder Informed Approach Underpinned by Implementation Science

To design the study procedure and intervention bundle, a scoping review has been completed to identify effective, evidence-based elements of a pharmacist-led TOC service [1]. This review found that pharmacist-led TOC services incorporating diverse inpatient and postdischarge activities improve medication safety and care coordination for patients with cardiovascular disease. Moreover, tailoring TOC services to specific patient populations and targeting care toward high-risk patients were important aspects to consider for pharmacist-led TOC services to support improved patient outcomes.

The findings from the scoping review were combined with feedback from key stakeholders (clinicians and consumers) to understand their needs when transitioning from the hospital. This approach ensured an evidence-based and contemporary approach to delivering an effective service to prevent readmission [1]. The objective of the stakeholder engagement was to understand current practice (positive and negative elements) and ascertain perspectives from hospital-based and primary care providers pertaining to the coordination of health care both during and after hospitalization. Local requirements for the TOC service were also gauged, with a focus on optimal medication management and exploring patient needs to incorporate within the new model. To ensure a culturally informed intervention, our research team has worked closely with the Institute for Urban Indigenous Health, a community-controlled health organization servicing clinics across the Greater Brisbane region, and formed a reference group comprised of Indigenous community members and Institute for Urban Indigenous Health staff.

The data gathering, mapping, and reference group formation have used a combination of focus groups, semistructured interviews, and yarning, as well as service and patient journey mapping. Applying Bradshaw’s approach, the study assessed the 4 “needs“ below [24]:

- Expert need: The gap between current practice provided at the hospitals when compared to the evidence for effectiveness when tailoring a TOC service to ensure that it is culturally appropriate and accessible. This has been identified in the scoping review and stakeholder focus groups [1].
- Expressed need: The way patients engage with the services or hospitals in their community.
- The felt needs of health care providers: The experiences and expectations of hospital health care providers, community pharmacists, and GPs (family doctors) to caring and discharging of this patient group.
- The felt needs of high-risk patients with cardiovascular disease: These are the care and knowledge gaps identified, and the experiences, expectations, and preferences reported by patients with cardiovascular disease about their hospital and postdischarge experiences.

Further details of phase 1 of this study are published elsewhere [25]. The bundle of care arising from the stakeholder involvement stages has informed the individualized care provided within the TOC service.

##### Phase 1 Part B: Risk Prediction Modeling

Risk prediction models to identify the risk of hospital readmission have been increasingly used in research to optimize care delivery and derive clinical benefit. In the AMI-CS cohort, models have been developed to predict 90-day readmissions with reasonable predictive performance [26,27]. However, these models omit medication-specific variables, and given the poor outcomes associated with early readmission, there is a need to develop a model to identify 30-day medication-related readmissions.

###### Aim

To develop and validate a risk prediction model to identify patients at risk of unplanned medication-related 30-day readmissions after an AMI and/or CS.

###### Methods

The model will be developed and validated using pretrial data from 1 to 2 Queensland hospitals. One thousand patients with a primary diagnosis of AMI and/or CS admitted to the PAH or TUH from July 2023 to June 2024 will be included in the model development study. Learnings from the development of a prior risk prediction model for medication harm [28] will be used to inform the new risk model.

Data will be collected from digital systems on patient characteristics, comorbidities, length of hospital stay, clinical parameters, and reason for admission. Other clinically relevant criteria not currently known will be identified through our consolidation of the literature [1] and data from part A.

###### Outcome

Medication-related hospital readmissions within 30 days of hospital discharge will be identified through a comprehensive retrospective review of patient clinical records by 2 senior pharmacists. Causality, preventability, and severity of potential medication-related hospital readmissions will be reviewed and rated by a panel of clinicians, including a cardiologist, nurse practitioner (NP), and pharmacist.

###### Statistical Analysis

Model evaluation will be guided by best practice guidelines, such as TRIPOD (Transparent Reporting of a Multivariable Prediction Model for Individual Prognosis or Diagnosis) statement [29], and the framework proposed by Steyerberg and Vergouwe [30] for developing robust prediction models.

Assuming an estimated medication harm readmission rate of 7% and the inclusion of 4 to 8 variables in the final model, a minimum events per variable ratio of 5 to 10 would suggest that at least 570 patients will be required, of whom approximately 40 will have medication harm readmission events.

Each variable will be evaluated using standard descriptive statistics and assessed visually to determine if it meets the basic assumptions for regression analysis. Missing data for each variable will be calculated, and where necessary, imputation methods will be used to conduct the analysis. Variables with more than 20% missing data will be excluded from the analysis. Univariable binomial logistic regression will be used to further assess the viability of candidate variables for multivariable analysis. Following this, we will fit a multivariable logistic regression model using an optimal subset of the candidate variables selected through either a penalized regression technique, such as LASSO (Least Absolute Shrinkage and Selection Operator) or elastic net [31,32], or by direct selection based on clinical relevance.

Predictive performance will be measured using the area under the receiver operating characteristic curve, as well as the area under the precision-recall curve. Sensitivity, specificity, positive predictive value, and negative predictive value will also be assessed at a cutoff chosen to classify the proportion of patients who will receive the intervention as “high-risk.” These metrics will be tested through cross-validation and reported for the best-performing model(s). Brier scores and calibration plots will be used to evaluate model calibration.

A deployable version of the optimally predictive model will be developed for use by clinical staff during phase 2 of the study. Patient data will be input by clinical staff, who will then be shown a prediction of the patient’s risk of medication-related readmission. The performance of the model will also be assessed at the end of phase 2.

### Phase 2: Pre-Post Interventional Study (RECARD Trial)

**Figure 1.**
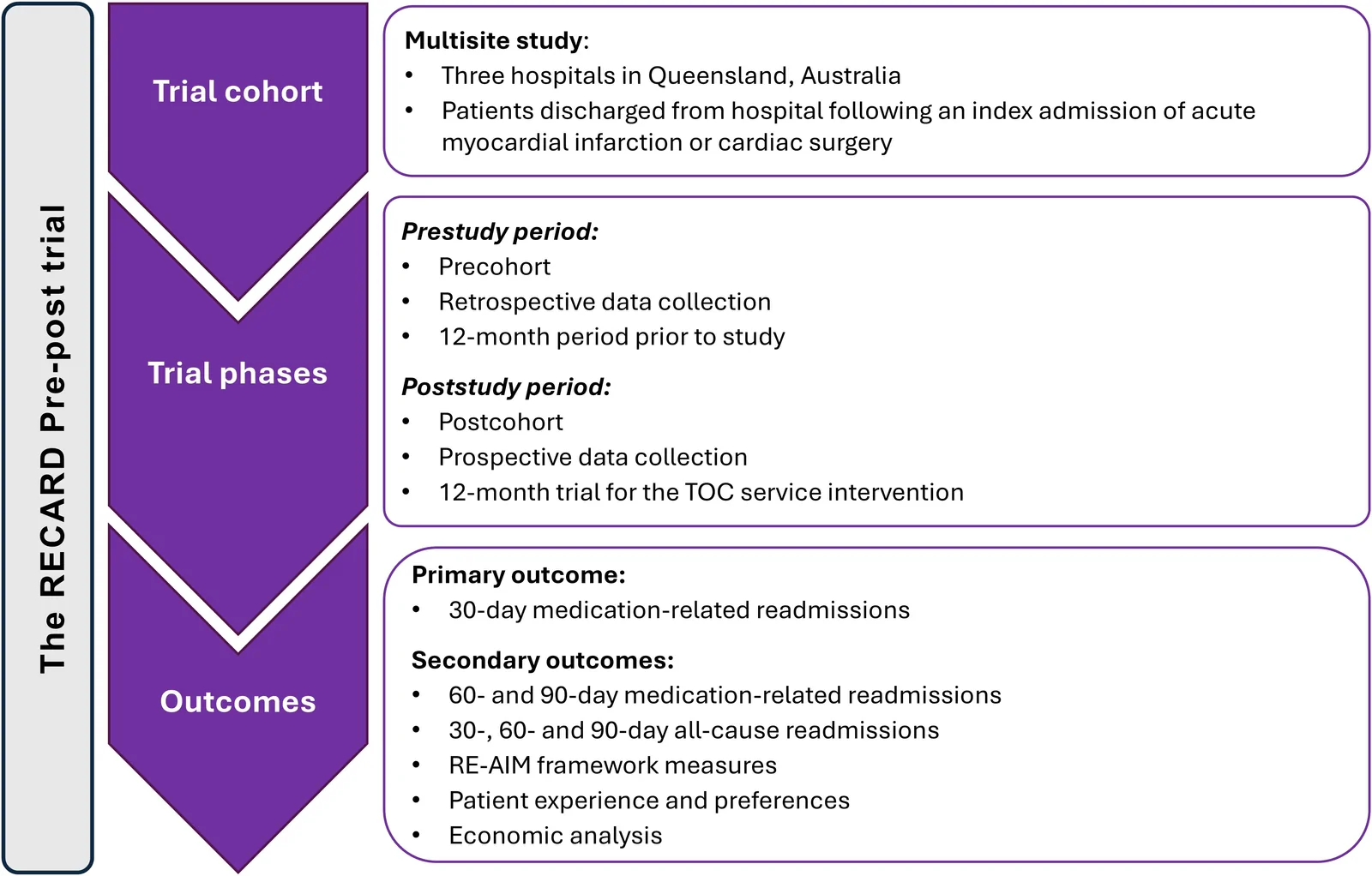
The trial schema outlining the RECARD (Reduce Hospital Readmissions for High-Risk Cardiology Patients) pre-post trial. RE-AIM: Reach, Effectiveness, Adoption, Implementation, and Maintenance; TOC: transition of care.

**Figure 2.**
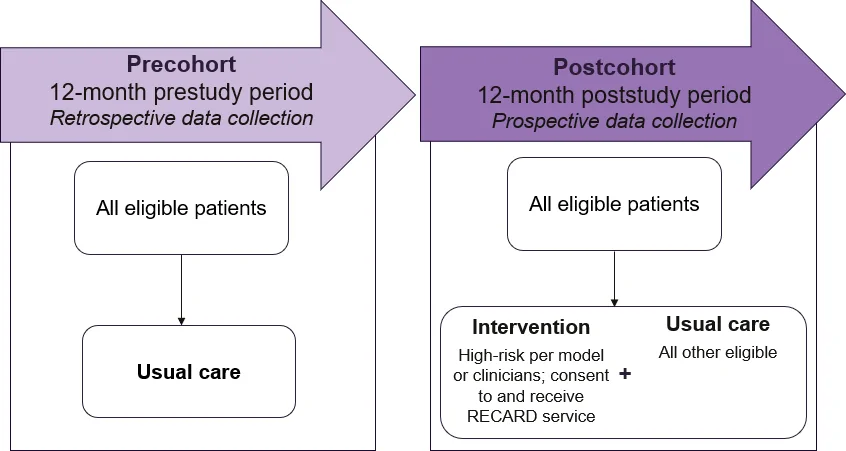
Pre-post study diagram showing the 2 study periods and outlining participants who will receive the study intervention vs usual care. RECARD: Reduce Hospital Readmissions for High-Risk Cardiology Patients.

This study will use a pre-post study that compares a retrospective historical cohort that received usual care and without a TOC service (precohort) to a prospective cohort that includes patients who receive either usual care or a pharmacist-led interdisciplinary TOC service (postcohort; Figures 1 and 2). During the poststudy period, patients identified as high risk will receive the individualized TOC service. These patients will be determined as those at the highest risk of medication-related hospital readmissions and will be termed “high-risk patients.” The rationale for delivering the TOC service intervention to high-risk patients is to ensure that those at greatest need (ie, risk of medication harm and readmission) are prioritized and thereby supporting long-term service sustainability.

The current local funding models do not allow every patient to receive an individualized service. We need to ensure a sustainable model of care that has the potential to be implemented and maintained within the Australian health care setting.

### Precohort

All patients admitted to the study sites with an index admission for AMI or CS between July 1, 2023, and June 30, 2024, will be included. A waiver of consent has been granted to collect these retrospective data for the historical usual care control group.

### Postcohort

All patients admitted to the study sites with an index admission for AMI or requiring CS between December 2025 and November 30, 2026; the period when the interdisciplinary pharmacist-led TOC service will be implemented.

### Eligibility Criteria

The inclusion criteria are as follows:

- Aged ≥18 years
- Discharged from PAH, RBWH, or TUH for an index diagnosis of AMI, or discharged from PAH or TUH for a CS admission
- Willing and able to provide informed consent

The exclusion criteria are as follows:

- Patients who are palliative or receiving end-of-life care
- Patients undergoing chemotherapy, radiation therapy, or dialysis
- Patients transferred to nonstudy hospitals
- Patients who discharged against medical advice
- Patients who died during their admission
- Patients admitted with type 2 myocardial infarction
- Patients who reside outside of Queensland
- Previous RECARD patients returning with a new AMI or CS event

The intervention period will require prospective collection of data for patients in the intervention group, who will be consented before receiving the TOC service. Patients who receive usual care have a waiver of consent to collect inpatient data and readmission data (Figure 2).

### Participant Identification, Recruitment, and Consent

Patients in the prestudy cohort will be retrospectively identified using the Health Information Management System reporting information and *International Statistical Classification of Diseases and Related Health Problems, Tenth Revision* (*ICD-10*; or equivalent) data. In contrast, patients in the postcohort will be identified through the presenting diagnosis (AMI or CS) or through a confirmed diagnosis on completed angiograms as per clinical records.

In the poststudy cohort, only patients identified as high-risk of readmission, either by the risk prediction model or by referral from the treating team, will be offered the TOC service model. Patients who are not identified as high-risk will receive usual care.

Patient recruitment will be completed by the TOC pharmacist or a member of the research team after patient identification by a member of either the TOC team, the research team, or the local treating team.

### Identification of “High-Risk” Patients

High-risk patients will be identified using 2 methods:

1. A risk prediction model (phase 1: part B) to identify patients who are at high-risk of medication-related readmission.
2. Identification by the cardiology treating teams and the cardiology ward pharmacist, who refer patients to the TOC service.

### RECARD Intervention: Pharmacist-Led TOC Service

Following patient consent, the TOC team will deliver assessments, activities, and medication reviews during the patient’s inpatient stay and for the 30 days postdischarge. The timeline for participant enrollment and assessments is shown in Table 1, created per the SPIRIT guidelines [23].

**Table 1.** Participant timeline for enrollment and assessments during the trial period.

Time point	Enrollment	Intervention			Closeout
	During hospital admission	Discharge from hospital	Day 3 postdischarge	Day 14 postdischarge	Day 30 postdischarge
Enrollment					
Eligibility screen	✓				
Informed consent	✓				
Assessments					
Adherence assessment		✓		✓	
Pharmacist activities			✓	✓	
Nurse practitioner consult				✓	
Patient experience survey					✓

The RECARD activities will be described in an intervention bundle that is provided to support the decision-making capabilities of the TOC clinicians. Furthermore, this bundle is intended to empower TOC clinicians to make critical judgments when tailoring the TOC service to individual patients (eg, not repeating tasks that have been performed by ward pharmacists or not offering services that would be foreseeably ineffective).

The bundle will be developed based on previous work by the research team, including findings from a scoping review [1], local studies exploring TOC at the participating sites [33], and qualitative studies with clinicians and patients as part of the larger RECARD program (HREC/2023/MNHB/104151 [25]).

**Figure 3.**
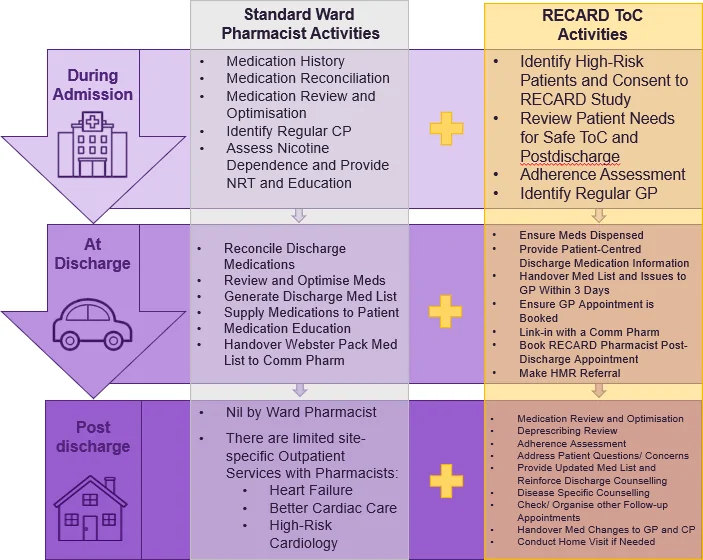
RECARD TOC study interventions across hospital transitions of care. CP: community pharmacy; HMR: home medicine review; GP: general practitioner; NRT: nicotine replacement therapy; RECARD: Reduce Hospital Readmissions for High-Risk Cardiology Patients; TOC: transition of care.

The work conducted in phase 1 seeks to inform the development of this bundle (see phase 1) [1]. The TOC service will be tailored to patients based on an assessment and identification of their individual needs and delivered during the hospital admission and across care transitions (Figure 3).

### Description of the TOC Service Model and Activities in the Bundle of Care

TOC services have many steps at which communication between patients and clinicians can occur (Figure 3). Table 1 highlights the potential touchpoints in the patient journey where various activities are provided to intervention participants as part of the TOC service. Typical activities delivered throughout a patient’s inpatient stay and in the postdischarge setting are described below [34]. The clinical skill set of NPs allows them to modify medication dosing, provide pathology requests, undertake patient observations, and interpret patient symptoms. In collaboration with the TOC pharmacist, the NP will run a 1-day-per-week clinic for patients who have specific comorbidities, pathology, physiology, or electrolytes requiring follow-up and patients with newly prescribed, withheld, or dose changes to β-blocker and angiotensin-converting enzyme inhibitor/angiotensin receptor blocker medications.

### Medication Education

Medication education is defined as the provision of tailored counseling (ie, having a conversation with the patient and/or their caregiver about their medications) delivered by a health care professional [35]. This includes informing the patient of any changes to their medication regimen (ie, ongoing medications, new medications, changed medications, or discontinued medications). The provision of medication education can occur at any time point across the continuum of a patient’s health care [36]. Additionally, this TOC service will ensure that all written medication lists provided to the patient are communicated to both the patient’s GP and community pharmacy after their education with the RECARD pharmacist.

### Disease-Specific Education

The TOC pharmacist, in liaison with a NP, will offer to provide lifestyle and, where required, disease-specific education, which may include counseling on modifiable risk factors such as diet, exercise, and smoking. This may also include the initiation or continuation of smoking cessation programs or nicotine replacement therapy, where appropriate. Disease-specific activities provided by the team may include further education on aspects such as symptom monitoring that are relevant to the patient’s cardiovascular condition (eg, salt intake or weight monitoring).

### Adherence Assessment and Provision of Adherence Aids

The TOC pharmacist will conduct a medication adherence assessment using 2 validated self-report tools, including the Medication Adherence Report Scale-5 [37,38]. An adherence assessment will be conducted as an inpatient activity (baseline) and as a postdischarge activity (follow-up). The adherence assessment will be used to tailor the information provided and identify interventions implemented for each individual identified as being at high risk of medication-related problems. If patients are assessed as nonadherent to their medications, the TOC team can implement an appropriate strategy to support medication adherence.

### Hospital Discharge Planning and Completion

The team engages in a collaborative decision-making process to determine a discharge plan and identify when the patient is ready for discharge. If the patient has consented to the TOC service, the ward pharmacist will provide a handover of all key issues and care provided for the patient during hospitalization to the TOC pharmacist. The TOC pharmacist must review prescriptions for clinical, ethical, legal, and Pharmaceutical Benefits Scheme requirements in accordance with relevant guidelines and standards. The TOC pharmacist will reconcile the medications prescribed at discharge. To complete the discharge process, the TOC pharmacist will also complete a discharge medication record (DMR), which is a list that encompasses a written description of instructions for discharge medications, a record of medication changes, and actions or updates for the patient and the primary care provider. Completion of the patient’s discharge also involves reviewing the discharge plan and undertaking any required actions, organizing prescriptions, and ensuring medication supply, if needed.

### Handover to Primary Care Provider

The TOC team will provide the DMR and other relevant discharge information to the primary care provider (GP and/or community pharmacist) using written and, potentially, verbal communication over the phone.

### Postdischarge Follow-Up Consults and Medication Optimization

The TOC team will organize a follow-up consultation with the patient through the platform of their preference (ie, in person, by video, by phone call, or through a home visit) and will confirm a date and time for this consultation within 14 days postdischarge (maximum 30 d). The follow-up consultation will encompass activities described above, such as medication review and optimization, medication education, and lifestyle education tailored to the patient. An updated medication record (same format as the DMR) will be provided to the patient if any changes have been made to their medication regimen since discharge. The TOC team will answer any questions or concerns, resolve any medication issues that arise during the follow-up consultation, and consolidate the medication education provided on discharge, if relevant. The TOC team will refer any issues to the treating team or primary care provider where appropriate. Home visits provide a unique opportunity to review a patient’s medicine stocks and storage practices. While the RECARD TOC team hopes to undertake some home visits where appropriate, this will not be possible for all patients requiring a home visit, especially those who live outside the hospital’s local area. For these patients, the TOC pharmacist will arrange a hospital-initiated Home Medicine Review referral. The Home Medicines Review pharmacist and TOC pharmacist will liaise to ensure the appropriate management of medication-related issues.

### Culturally Safe Care

The TOC team will aim to provide culturally safe care to all patients, guided by information collected in the yarning circles conducted in phase 1. For patients identifying as Aboriginal and/or Torres Strait Islander, this care will be guided and supported by an Indigenous health worker. The Indigenous health worker will yarn with Aboriginal and Torres Strait Islander patients as needed to help identify the challenges they are experiencing and engage them with the pharmacist, NP, and rehabilitation programs.

### Usual Care

Usual care across the hospital sites includes nurse and pharmacist involvement during admission and at discharge (as resources permit), according to standard operating procedures. All patients who are not identified as being at high risk of readmission during the intervention period, along with all patients in the prestudy period (usual care control group), will receive usual care and will not be provided the interdisciplinary pharmacist-led TOC service. Usual care typically does not include dedicated pharmacy support as part of postdischarge follow-up.

### Outcome Measures

#### Primary Outcome: Medication-Related Hospital Readmissions and Causality Analysis

All patients who are readmitted to the hospital within 30 days following discharge from the PAH, RBWH, or TUH will be retrospectively reviewed to identify whether their readmission was medication-related.

For the primary outcome of 30-day medication-related hospital readmissions, cases will be confirmed and validated using a bimodal approach. The first will include the use of *International Statistical Classification of Diseases and Related Health Problems, Tenth Revision, Australian Modification* (*ICD-10-AM*) “Y” codes to determine “drugs, medicaments and biological substances causing adverse effects in therapeutic use.” The second approach will involve the review of the patient discharge summary, progress notes, and laboratory markers by 2 senior pharmacists to confirm 30-day medication-related readmission.

Finally, an expert panel comprising a senior pharmacist, specialist nurse, cardiologist, and researcher will independently review readmissions to reach a final consensus. The expert panel will confirm medication-related hospital readmissions by assessing causality using tools such as the Naranjo algorithm or the World Health Organization Uppsala Monitoring Centre criteria.

#### Secondary Outcomes

**Figure 4.**
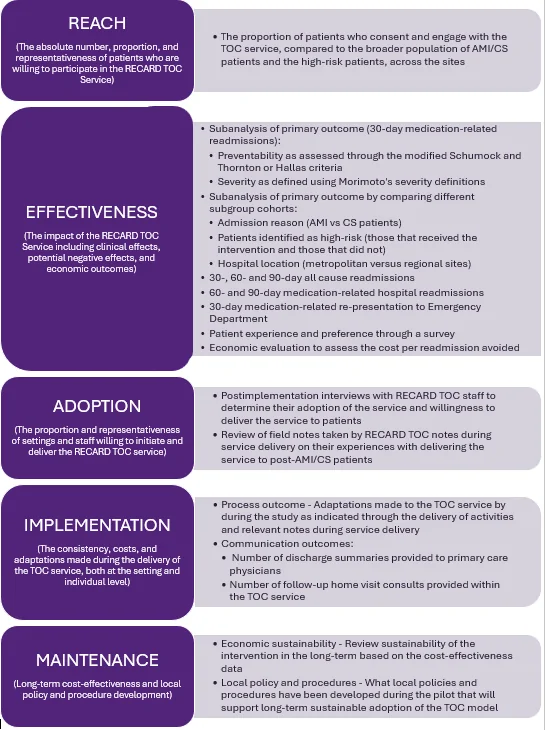
Mapping of secondary outcome measures in the RECARD trial using the RE-AIM (Reach, Effectiveness, Adoption, Implementation, and Maintenance) framework. AMI: acute myocardial infarction; CS: cardiothoracic surgery; RECARD: Reduce Hospital Readmissions for High-Risk Cardiology Patients; TOC: transition of care.

The secondary outcomes are summarized in Figure 4. The secondary outcomes have been chosen based on a hybrid effectiveness-implementation design to explore strategies to translate the intervention into practice and are mapped to the RE-AIM framework [20].

### Analysis Types of Harm

The severity and preventability of 30-day medication-related readmission will be assessed as a secondary outcome to assess the types of harm. Methods to assess preventability will include the use of the modified Schumock and Thornton criteria [21]. Methods to assess the severity of medication harm will follow the definitions described by Morimoto et al [22]. A multidisciplinary panel will also review preventability and severity of the medication-related hospital readmissions, as used for causality analysis.

### 60- and 90-Day Medication-Related Hospital Readmissions

*ICD-10-AM* “Y” codes to determine “drugs, medicaments and biological substances causing adverse effects in therapeutic use” will be used to identify 60- and 90-day medication-related hospital readmissions.

### Patient Experience Survey

A patient survey will be developed and administered at the end of the trial to assess patient experience and elicit patient preferences using a discrete choice experiment (DCE). Patient experience will be assessed with a series of multiple-choice, short-answer, and Likert scale questions. Adjustments will be made to the survey after piloting, and the final survey will be uploaded into Qualtrics. The DCE will be conducted to determine the relative importance of each element of the intervention to the patient population and the associated willingness to pay. DCEs use random utility theory to estimate the value of health service provision based on individual attributes of the service [39]. They involve asking the consumers to make discrete choices between mutually exclusive options containing different levels of predefined attributes. The survey will be administered to all patients in the trial where possible.

### Economic Evaluation

Cost-effectiveness of the pharmacist-led interdisciplinary TOC service, compared to usual care, for patients post-AMI and/or CS will be determined using data from the primary analysis. A cost-effectiveness analysis will be undertaken to determine the net cost of providing the intervention and any difference in the cost of unplanned 30-day readmissions (primary outcome) compared with the usual care group. Costs will be quantified using data from the trial, the local hospital business unit, and expert opinion. Effectiveness will be measured as the incremental reduction in unplanned 30-day readmissions, and this will be used to determine the incremental cost-effectiveness ratio. All costs will be defined from the perspective of the state health system for a single year (the financial year of the trial) and currency (Australian dollars, AUD). *P* values less than .05 will be considered statistically significant. The analysis method may be adapted to a cost-minimization analysis or cost-consequence analysis, if required, once the primary outcomes have been analyzed. Sensitivity analysis will be conducted to assess the level of confidence in the conclusions of the economic evaluation. Sensitivity analysis is expected to include variations in resource costs, hospitalization costs, remuneration and savings from the program, and outcome measures, but specific attributes will be identified and their variation limits will be determined after the primary cost-effectiveness analysis has been completed.

The implementation and maintenance secondary outcomes will be evaluated using quarterly interviews with the TOC team to identify site-specific adaptations as well as enablers and barriers to TOC delivery.

### Sample Size

It is expected that, under conditions of usual care, the rate of medication-related hospital readmissions within 30 days among patients discharged following an AMI is 7% [40]. Interventions have reduced this rate from 7% to 4.9% (an absolute risk reduction of 2.1%) [41]. As mentioned previously, only a subset of the population (those identified as being at highest-risk based on model scoring and/or clinician referral) will be targeted to receive the intervention. Guided by previous studies and resources, we expect to prioritize the top 25% of patients at high-risk of medication-related readmission [42]. An additional 10% of patients are expected to be referred by the treating team. However, the final proportion of patients who will be triaged for intervention will be guided by a local audit following the development of the risk model. Because the high-risk patients will likely have a higher rate of potential readmission (ie, >7%), we expect that an intervention applied to only this group will still produce an absolute risk reduction of at least 2.1% averaged across the entire intervention sample, even though we expect the risk in the low-risk group to remain unchanged. Therefore, the sample size calculation is based on the overall sample required, not just the high-risk group.

Assuming a baseline risk of readmission of 7% and a significance level of .05, a sample size of 1990 participants is required in both the prestudy and intervention periods (ie, a total of 3980 patients across both prestudy and intervention periods) to detect an absolute risk difference of 2.1% with a power of 0.80. We assume a minimal dropout and refusal rate of 10%; taking this into account, we aim to recruit 2211 participants into each 12-month period (a total of 4422 participants across both the prestudy and intervention periods). We expect 35% of patients (~775 patients) to be flagged as high risk, consent to the study, and be given the TOC intervention. The remainder of the patients enrolled in the study will be given usual care as described earlier. Delivering the TOC service to 775 participants across the study sites over 12 months is both logistically feasible and achievable.

### Statistical Analysis

An intention-to-treat analysis will be conducted. Participants who are lost to follow-up (ie, participants who do not complete the intervention, withdraw consent, or die during the study) will be included in the intention-to-treat analysis for the primary outcome. Participant characteristics will be summarized according to the prestudy and poststudy cohorts. Characteristics will be summarized and presented descriptively using mean (SD), median (IQR), and n (%), as required. Independent *t* tests and chi-square tests will be undertaken to determine whether there are any differences in baseline characteristics between the prestudy and intervention cohorts.

The primary outcome is a comparison of 30-day medication-related hospital readmissions in the prestudy cohort compared with the poststudy cohort. Secondary outcomes will be compared similarly. The analysis will consist of multivariable logistic regression. The explanatory variables will consist of a variable indicating the study period along with any relevant potential confounding variables we wish to control for (eg, patient age, sex, etc). The results for these outcomes will be considered significant at the 5% level, with 95% CIs reported. Subgroup analysis will also be undertaken to assess whether the clinical impact of the intervention varied by location (regional vs metropolitan sites), Indigenous and non-Indigenous patients, and high-risk patients identified according to the model of treating team referral.

Evaluation will be conducted using the RE-AIM framework as described earlier [20]. Data will be analyzed descriptively, and other metrics (eg, primary outcome) will be mapped as calculated above or informed by the patient survey and economic analysis.

### Data Collection

Patient data will be collected and collated by the research assistants and postdoctoral research fellows using standardized electronic (REDCap) data collection forms.

### Precohort (Retrospective Data Collection)

Data will be collected from Health Information Management System to identify patients who are readmitted within 30 to 90 days. Re-presentation data for the ED will require data collection from Cerner FirstNet, as *ICD-10* codes are not used at the ED sites of the participating hospital. Sociodemographic characteristics, such as age, sex, height, weight, and Aboriginal and/or Torres Strait Islander status; clinical characteristics, such as current medications, medication history, comorbidities, laboratory results (ie, full blood count, renal function tests, liver function tests, coagulation studies, lipid levels, etc), and the Charlson Comorbidity Index; and hospitalization characteristics, such as length of stay, presenting complaint, ward location, cost of admission, and any associated events (readmissions, hospital-acquired complications, or other), and treating team will be compiled using available electronic sources and digital health records. These data will be extracted using hospital reporting systems such as the Hospital Based Corporate Information System and the integrated electronic medical record.

### Postcohort (Prospective Data Collection)

Research staff will use standardized data collection forms to collect the activities that were delivered to patients during the intervention. The same variables will be collected in both the preperiods and postperiods to enable direct comparison of results. Collected data will include sociodemographic, clinical, hospitalization characteristics, and costs associated with the admission of interest and any associated events (readmissions, hospital-acquired complications, or other).

### Data Management

Data will be collected at the sites using standardized electronic (REDCap) data collection forms. REDCap data are encrypted in transit and storage and require 2-factor authentication. REDCap is a secure application that will enable data sharing between Queensland Health (QH) sites. Data will also be stored electronically in a safe and secure manner on a password-protected secure drive on the QH server and/or on the University of Queensland (UQ) Research Data Manager (RDM) server. Deidentified data and statistical analyses can be accessed with appropriate ethics and governance approvals through the UQ RDM system.

The UQ RDM is an access-controlled, authenticated network computer drive managed by Information Technology Services. Trial data will be stored after completion for a minimum of 7 years.

### Trial Monitoring

The RECARD program has a Program Steering Committee (PSC) and an Expert Advisory Board (EAB) to which any issues or concerns can be escalated from the research team. In the event of any potential adverse events, these will be managed at each site as per their local care procedures and within QH guidelines. The trial has also been registered, and a review of safety reports will be maintained as a standing agenda item for the PSC meetings so that any questions or concerns can be raised regularly and consistently. To enable optimal safety and harm reporting, there will also be 3 external clinicians who are independent of the RECARD trial team, the PSC, and the EAB, who will act as safety monitors. They will be provided with readmission reports for the intervention period related to cardiac readmissions to each hospital. Trends for readmissions (starting 3 months prior to implementation of the new service model [poststudy]) will be monitored, and any rise potentially attributable to the new model of care will be further investigated at the relevant site.

### Organization Structure of the Trial

The Program Management Committee (PMC) will oversee the trial on behalf of the sponsor (the sponsor retains ultimate responsibility for the trial). The PMC is focused on operational matters regarding the trial management. The PMC will provide regular reports to the PSC, such as information on study status, protocol compliance, budget, data management, legal or regulatory issues, and risk management and mitigation.

The PSC will oversee the conduct and integrity of the RECARD trial and will provide support to the PMC. The PSC is responsible for the execution of all activities, including trial design, finalization of the protocol, intervention delivery, data collection, data management, data analysis, report writing, milestone reporting, and results dissemination. An important function is to support risk management and provide status reports to the EAB.

The EAB comprises international experts who will provide oversight and advice to the PSC in relation to the RECARD trial. The UQ School of Pharmacy and Pharmaceutical Sciences is the coordinating faculty on behalf of UQ.

### Ethical Considerations

#### Ethical Approvals

All projects within this study have received ethical approval from the following Human Research Ethics Committees (HRECs). Phase 1A of this project has received ethical approval from the Metro North HREC (HREC/2024/MNHB/103581 and HREC/2024/MNHB/104151) and the UQ HREC (2025/HE001167). Phase 1B of the project has received ethical approval from the TUH HREC (T/2024/QTHS/107694). Phase 2 of this project has received ethical approval from the Metro South Health HREC (HREC/2024/QMS/109242).

#### Informed Consent

Participants recruited to the TOC service in the prospective cohort of the trial (phase 2) will be provided with information about the trial and will need to provide written or verbal consent prior to receiving any intervention.

Consent for publication will also be obtained from these study participants, which will include consent to publish aggregated deidentified data in a relevant medical journal.

Consent for the remaining participants in the prospective and retrospective cohorts of the RECARD trial (phase 2), who receive usual care, is covered by Public Health Act (PHA) approvals from the Queensland Government (PHA 107694 and PHA 109242).

#### Privacy and Confidentiality

Only deidentified data from participants will be published.

#### Participant Compensation

Participants in the stakeholder engagement aspects of this project (part 1A) received compensation in the form of vouchers at the end of their involvement.

#### Dissemination of Findings

The results will be disseminated via reports to the hospitals involved, their governing bodies, and key stakeholders, including the UQ and the Medical Research Future Fund. In addition, our findings will be disseminated via academic papers, presentations at scientific conferences, and a general report of key findings and recommendations for a broader rollout of cardiac transition-of-care activities in Australian hospitals.

## Results

Phase 1 of the RECARD Program involved focus groups with clinicians and patient interviews, and these have been completed and analyzed, and the findings have informed our main trial. For the main trial (RECARD), the study has commenced, with recruitment commencing in December 2025. A risk tool has been developed using retrospective data and is being used to identify eligible high-risk patients for recruitment. As of May 27, 2026, a total of 343 patients have been recruited. The PAH has recruited 159 patients, the TUH has recruited 131 patients, and the RBWH has recruited 53 patients. Recruitment will continue until November 2026. Analysis will commence shortly thereafter (currently planned for January to March 2027). No preliminary analysis has been undertaken.

## Discussion

### Impact

Implementation of an interdisciplinary pharmacist-led cardiology TOC service has the potential to significantly reduce 30-, 60-, and 90-day medication-related hospital readmissions for patients following AMI or CS. It can also lead to reductions in all-cause readmissions at 30, 60, and 90 days, improved patient-reported experiences, and be cost-effective.

### Multifaceted Interventions to Reduce Medication-Related Hospital Readmissions

Several pharmacist services show clinical and cost-effectiveness, for example, a best possible medication history, reconciliation, and clinical review [43]; discharge education and postdischarge follow-up [1,44]. However, the process of tailoring a series of individualized interventions using a multidisciplinary team in patients with AMI is novel to this study. The benefit of multidisciplinary postdischarge interventions led by pharmacists in other specialties, such as geriatric and general medical patients, has been reported [45]; however, this has not been investigated in high-risk cardiac patients.

### Targeting High-Risk AMI/CS Patients

Risk prediction models are rapidly evolving, particularly within digitally enabled hospitals, where increasing data availability creates opportunities to improve patient stratification and care delivery. In the context of constrained health care resources, accurately identifying patients who are most likely to benefit from interventions is essential to optimize resource allocation and reduce avoidable harm. Established models, such as the LACE (Length of stay, Acuity of the admission, Charlson Comorbidity Index, and Emergency department visits) index for hospital readmissions [46] and other tools developed for general cardiac populations for all-cause readmission [47], demonstrate the value of prediction approaches in guiding clinical decision-making and targeting high-risk groups.

However, there is currently no dedicated model tailored to patients following AMI or CS, nor is there one that specifically predicts medication-related readmissions in this population. Developing such a model is particularly important given the complexity of post-AMI pharmacotherapy and the associated risk of adverse medication events. Importantly, the predictive performance of risk prediction models is strongest when they are derived from cohorts closely aligned with the intended clinical population, reinforcing the need for AMI-specific tools. As such, implementing a robust, context-specific risk prediction model could enable clinicians to proactively identify high-risk patients and target interventions to prevent medication-related harm after AMI.

### Limitations

A key limitation of this pre-post study design is the potential failure to achieve the intended recruitment target in the postintervention phase, for which there are no clear mitigation strategies. This may reduce statistical power and limit the ability to detect meaningful differences between periods. However, to date, the study is progressing well with recruitment. Additionally, the retrospective-prospective nature of the design involves comparing different patient cohorts across separate time periods, meaning that the groups are not strictly comparable. Temporal changes in patient characteristics and clinical practice may confound the results, making it difficult to attribute observed differences solely to the intervention. This risk can be managed as some confounders can be accounted for or adjusted for in our statistical analysis. A controlled cohort design could have improved comparability and reduced bias; however, such an approach was not feasible in this context. Specifically, withholding a gold-standard medication management service from high-risk patients—who are most likely to benefit—would raise ethical concerns, necessitating the use of a less rigorous but more ethically appropriate pre-post design.

The study is among the first to determine whether a pharmacist-led Cardiology TOC service reduces 30-day medication-related patient readmissions and secondary outcomes. The findings will inform future implementation of a sustainable cardiac TOC model within the Australian health care setting.

The recent publication of the Medication Management at Transitions of Care Stewardship Framework by the Australian Commission [48] emphasizes that TOC, especially from the hospital to the home, is a high-risk period for medication errors, miscommunication, and preventable harm and therefore requires a structured stewardship approach to improve safety and outcomes. It outlines 4 core elements: strong governance through a designated committee, a multidisciplinary stewardship team to lead and champion improvements, coordinated medication management activities from admission to postdischarge, and continuous monitoring, evaluation, and feedback. The framework highlights person-centered care, effective communication, and digitally enabled information sharing as essential enablers, stressing early risk assessment, clear roles between providers, medication reconciliation at every transition, and robust, timely discharge communication [48].

The RECARD study follows these guidelines to provide a systematic, scalable model for health services to strengthen medication safety, reduce medication-harm-related readmissions, and ensure safer, more seamless care across the entire patient journey.

## Supplementary material

10.2196/94841 Checklist 1 SPIRIT checklist.

## Abbreviations

AMI: acute myocardial infarction
CS: cardiothoracic surgery
DCE: discrete choice experiment
DMR: discharge medication record
EAB: Expert Advisory Board
GP: general practitioner
HREC: Human Research Ethics Committee
*ICD-10*: *International Statistical Classification of Diseases and Related Health Problems, Tenth Revision*
*ICD-10-AM*: *International Statistical Classification of Diseases and Related Health Problems, Tenth Revision, Australian Modification*
LACE: Length of stay, Acuity of the admission, Charlson Comorbidity Index, and Emergency department visits
LASSO: Least Absolute Shrinkage and Selection Operator
NP: nurse practitioner
PAH: Princess Alexandra Hospital
PHA: Public Health Act
PMC: Program Management Committee
PSC: Program Steering Committee
QH: Queensland Health
RBWH: Royal Brisbane and Women's Hospital
RDM: Research Data Manager
RE-AIM: Reach, Effectiveness, Adoption, Implementation, and Maintenance
RECARD: Reduce Hospital Readmissions for High-Risk Cardiology Patients
SPIRIT: Standard Protocol Items: Recommendations for Interventional Trials
TOC: transition of care
TRIPOD: Transparent Reporting of a Multivariable Prediction Model for Individual Prognosis or Diagnosis
TUH: Townsville University Hospital
UQ: University of Queensland
